# Evaluation of sex differences in patients with ST-elevated myocardial infarction: an observational cohort study in Amsterdam and surrounding region

**DOI:** 10.1007/s12471-020-01435-9

**Published:** 2020-06-11

**Authors:** T. Kerkman, L. B. G. ten Brinke, B. Huybrechts, R. Adams, G. Amoroso, R. J. de Winter, Y. Appelman

**Affiliations:** 1 Department of Cardiology, Amsterdam UMC, location VUmc, Amsterdam, The Netherlands; 2 Emergency Medicine Services Amsterdam, Amsterdam, The Netherlands; 3 Department of Cardiology, Amsterdam UMC, location AMC, Amsterdam, The Netherlands; 4 Department of Cardiology, OLVG Oost, Amsterdam, The Netherlands

**Keywords:** ST-elevation myocardial infarction, Primary percutaneous coronary intervention, Sex differences, Amsterdam

## Abstract

**Introduction:**

Women with ST-elevation myocardial infarction (STEMI) present with different symptoms compared to men. This can result in delays in diagnosis and in the timely treatment of women. The aim of this study is to examine these differences, including the short- and long-term mortality in women and men.

**Methods:**

This quality registry study included all patients with STEMI who received primary percutaneous coronary intervention in 2015 or 2016 in Amsterdam and the surrounding region.

**Results:**

Three PCI centres and the Emergency Medical Service in Amsterdam participated. In total, 558 men (71%) and 229 women (29%) were included. Women were on average 7 years older than men (68 vs 61 years, *p* < 0.001), and suffered more often from hypertension (46% vs 34%, *p* = 0.002) and monovascular disease (69% vs 57%, *p* = 0.002). A higher percentage of men were current smokers (41% vs 49%, *p* = 0.043). Patient delay, system delay and overall ischaemic times were similar in both women and men (medians: 51, 94 and 157 min, respectively). Initiation of treatment was achieved within 90 min after STEMI diagnosis in 85% of patients (87% in women, 85% in men). Thirty-day and 1‑year mortality adjusted hazard ratio for women versus men was 1.60 (95% CI 0.9–3.0) and 1.24 (95% CI 0.8–2.0), respectively.

**Discussion:**

Recognition of cardiac complaints remains challenging for patients. In the Amsterdam region, time delays and mortality were not significantly different between men and women presenting with STEMI. These results are in contrast to findings in similar registries. This suggests that implementation of current knowledge and national campaigns are effective in increasing awareness of the signs and symptoms suggestive of myocardial infarction.

## What’s new?

- Patient delay, system delay and total ischaemic times are similar in female and male patients presenting with ST-elevated myocardial infarction (STEMI) who have received primary percutaneous coronary intervention (pPCI).
- In Amsterdam and the surrounding region, 85% of the patients presenting with STEMI receive pPCI within 90 min after diagnosis.
- Mortality rates following pPCI after STEMI are low and comparable in women and men in Amsterdam and the surrounding region.
- In almost 90% of patients radial access was achieved, with the percentage similar in both women and men.
- Acquiring information concerning complications in patients receiving pPCI is difficult but of upmost importance for quality purposes.

## Introduction

In 2004, a pre-hospital triage system was introduced in Amsterdam and the surrounding region with the aim of quickly identifying patients with ST-elevation myocardial infarction (STEMI) who are candidates for coronary reperfusion therapy. This method of diagnosis minimises pre- and in-hospital times to achieve coronary revascularisation as quickly as possible. In Europe, patient delay [1–3] and overall ischaemic time [4] are reported to be longer in women. Women experience higher in-hospital [2, 3, 5] 30-day [4] and 1‑year [6] death rates than men. In various parts of the Netherlands women have been shown to have increased total ischaemic times compared to men [7] and (young, [8]) women had increased mortality rates following STEMI when compared to similarly aged men [9]. In Amsterdam and the surrounding region this sex-specific information is lacking thus far. Therefore, the main goal of this study was to examine if sex-based differences exist in STEMI patients in Amsterdam and the surrounding region, focusing on patient delay, system delay and overall ischaemic time as well as all-cause mortality at 30 days and 1 year following primary percutaneous coronary intervention (pPCI).

## Methods

This study investigates data from the pre-hospital triage system focusing on STEMI patients receiving pPCI in Amsterdam and the surrounding area. Data inclusion comprises the period from January 2015 until December 2016. At that time, Amsterdam and the surrounding area had 1.31 million inhabitants [10], an area of 693 km^2^ [11], three pPCI-capable hospitals (Onze Lieve Vrouwe Gasthuis, Amsterdam UMC—location VUmc and location AMC), seven non-intervention hospitals and two ambulance services. As soon as dispatchers answering the emergency telephone suspect a myocardial infarction, the ambulance crew is instructed to drive to the patient. Based on the 12-lead electrocardiogram (ECG) recorded by the specialised nurse upon arrival, the cardiologist on call decides whether to accept the patient for pPCI and go directly to the catheterisation laboratory, bypassing any medical emergency department. Simultaneously the ambulance staff stabilises the patient and starts pharmacological treatment according to protocol, consisting of 160 mg acetylsalicylic acid orally or 500 mg Aspegic intravenously, 5000 IU unfractionated heparin intravenously and an oral loading dose of 180 mg ticagrelor. Following the procedure, patients are transferred to the affiliated non-intervention hospital if possible.

### Inclusion and exclusion criteria

The medical ethical/advisory scientific committee of each intervention hospital granted permission. Patients with an acute coronary syndrome identified as STEMI, according to the European Society of Cardiology guidelines [12], were included. Patients not receiving coronary angiography (CAG) were excluded, as well as patients receiving CAG/PCI later than 24 h after the first medical contact, since these patients were unlikely to have suffered a primary infarction.

### Data management

This data set includes 78 variables describing baseline characteristics, demographic variables, the exact time of consecutive events prior to PCI and peri-procedural characteristics. Three full-time dedicated staff research nurses and one dedicated ambulance organisation member gathered all the information. Thirty-day and 1‑year mortality is established by social security number.

### Time intervals and definitions

Several time intervals are calculated in minutes: patient delay, system delay and the overall ischaemic time. Patient delay is defined as the time interval from onset of symptoms until ambulance dispatch. System delay includes multiple time intervals: ambulance dispatch until reaching the patient and recording the first ECG, from STEMI diagnosis to arrival at the pPCI centre, from pPCI centre to arterial access and from arterial access to balloon inflation in the culprit artery. Total ischaemic time is the timespan from symptom onset until balloon inflation in the culprit artery. The baseline characteristics were interpreted according to the definitions of the National Institute for Public Health and the Environment [13] and are presented in the Appendix.

### Analysis

When not all the required information could be entered into the database, the unknown data was treated as a missing value. If less than 5% of a variable consisted of missing values, these data were excluded without further consequence. Since all data presented consisted of less than 5% missing values, no multiple imputation was used. Data are presented as numbers and percentages for categorical variables and as means (with standard deviation) for continuous variables. Different time intervals are presented as median and mean with interquartile range (IQR). Differences in baseline as well as procedural characteristics were analysed using the chi-square test, or Fisher’s exact test if appropriate. Median time intervals were compared by the independent samples test. Time intervals were log-transformed if not normally distributed, and the difference in mean between women and men was analysed by linear regression. Thirty-day and 1‑year mortality rates were calculated for both women and men. Estimates of the cumulative incidence of mortality were obtained using the Kaplan-Meier method, and intersexual differences were evaluated by the log-rank test. All tests in the analysis were two-sided. A *p*-value <0.05 was considered statistically significant. Statistical analysis was performed using IBM SPSS version 24.

## Results

In total, 3874 ECGs were analysed from patients suffering symptoms suspicious for STEMI, of which 787 patients fulfilled the inclusion criteria (Tab. 1). The population consisted of 229 women and 558 men, 29% and 71% respectively. Women were significantly older with a mean difference of 7 years (68 ± 14 vs 61 ± 12, *p* < 0.001). Regarding cardiovascular risk factors, hypertension was more common amongst women than men (46% vs 34%, *p* = 0.002) and women were less often current smokers (41% vs 49% *p* = 0.043). Out-of-hospital cardiac arrest (OHCA) was less prevalent in women, yet this difference did not reach statistical significance (8% in women vs 12% in men, *p* = 0.160). Pre-procedural administration of medical treatment consisting of platelet inhibitors or heparin was not significantly different between women and men.

**Table 1:** Baseline characteristics

	Women *n* (%) *N* = 229 (29)	Men *n* (%) *N* = 558 (71)	*p*-value
Age, years (mean ± SD)	68 ± 14	61 ± 12	<0.001
*Intervention hospital*			
AUMC, AMC	36 (15.7)	79 (14.2)	
AUMC, VUmc	98 (42.8)	255 (45.7)	
OLVG	95 (41.5)	224 (40.1)	
*Risk factors*			
Hypertension	101 (45.7)	178 (33.6)	0.002
Diabetes mellitus	39 (17.6)	66 (12.5)	0.065
Hypercholesterolaemia	56 (25.9)	110 (21.0)	0.144
Family history of cardiovascular disease	71 (33.0)	167 (32.4)	0.862
Current smoker	88 (41.1)	258 (49.3)	0.043
*Medical history*			
Previous MI	30 (13.6)	79 (13.7)	0.977
Previous PCI	33 (14.4)	77 (14.2)	0.850
Previous CABG	7 (3.1)	13 (2.4)	0.572
Previous CVA	15 (6.8)	20 (3.7)	0.063
Previous PVD	8 (3.7)	20 (4.0)	0.899
Previous CKF^a^	6 (2.8)	9 (1.7)	0.343
Previous CHF	6 (2.9)	5 (1.0)	0.057
*Pre-procedural characteristics*			
OHCA	19 (8.3)	65 (11.7)	0.160
Acetylsalicylic acid administered	213 (93.0)	525 (94.6)	0.392
Ticagrelor administered	189 (82.5)	459 (82.3)	0.914
Heparin administered	223 (97.4)	529 (95.5)	0.217
*Procedural characteristics*			
Time of procedure			
– Office hours (08:00–17:00 h)	93 (49.7)	239 (50.9)	0.796
– Evening hours (17:00–24:00 h)	44 (23.5)	104 (22.1)	0.698
– Night time (00:00–08:00 h)	50 (26.7)	127 (27.0)	0.941
Access			0.068
– Radial	192 (86.1)	482 (90.6)	
– Femoral	31 (13.9)	50 (9.4)	
Number of lesions			0.002
– Zero (*n* = 1) or one	155 (69.2)	311 (57.2)	
– Two or more	69 (30.8)	233 (42.8)	
Culprit lesion			
– LM	2 (0.9)	12 (2.2)	0.372
– LAD	82 (36.9)	438 (44.2)	0.076
– RCX	22 (9.9)	84 (15.6)	0.040
– RCA	116 (52.3)	204 (37.8)	0.000
– Graft	0 (0)	1 (0.2)	0.999
Number of stents			0.586
– Zero (*n* = 34) or one	152 (67.9)	380 (69.9)	
– Two or more	72 (32.1)	164 (30.1)	
DES used	202 (94.4)	501 (96.2)	0.286
Hypotension	16 (7.2)	29 (5.4)	0.321
TIMI grade flow pre-PCI			0.542
– TIMI 0	119 (54.3)	311 (57.6)	
– TIMI 1	18 (8.2)	48 (8.9)	
– TIMI 2	28 (12.8)	43 (8.0)	
– TIMI 3	54 (24.7)	138 (25.5)	
TIMI grade flow post-PCI			0.942
– TIMI 0	3 (1.4)	6 (1.1)	
– TIMI 1	1 (0.4)	6 (1.1)	
– TIMI 2	15 (6.8)	33 (6.2)	
– TIMI 3	201 (91.4)	488 (91.6)	

Data presented as *n* (%) if not otherwise indicated

*AUMC, AMC* Amsterdam University Medical Centres located at Academic Medical Centre, *OLVG* Onze Lieve Vrouwen Gasthuis, *AUMC, VUmc* Amsterdam University Medical Centres located at Vrije Universiteit, *N* number of patients, *MI* myocardial infarction, *PCI* percutaneous coronary intervention, *CABG* coronary artery bypass graft, *CVA* cerebrovascular accident, *PVD* peripheral vascular disease, *CKF* chronic kidney failure, *CHF* chronic heart failure, *OHCA* out-of-hospital cardiac arrest, *LM* left main artery, *LAD* left anterior descending artery, *RCX* right circumflex artery, *RCA* right coronary artery, *DES* drug-eluting stent, *TIMI* thrombolysis in myocardial infarction

^a^Estimated glomerular filtration rate <50 ml/min per 1.73 m^2^

### Procedural characteristics

Women presented more often with single-vessel disease (69% vs 57%, *p* = 0.002). In total, 498 patients received one stent (136 women, 362 men) and 236 patients (72 women, 164 men) received two or more stents. Thirty-four patients did not receive a stent, of which 5 patients died (4 women and 1 man). In total, 18 patients (13 men and 5 women) died on the day of the procedure, most likely due to complications such as arrhythmia or hypotension. Drug-eluting stents were used most often (94% in women and 96% in men, *p* = 0.286) and transradial arterial access was achieved in almost equal percentages of women and men (86% in women and 91% in men, *p* = 0.068).

### Time intervals

**Fig. 1:**
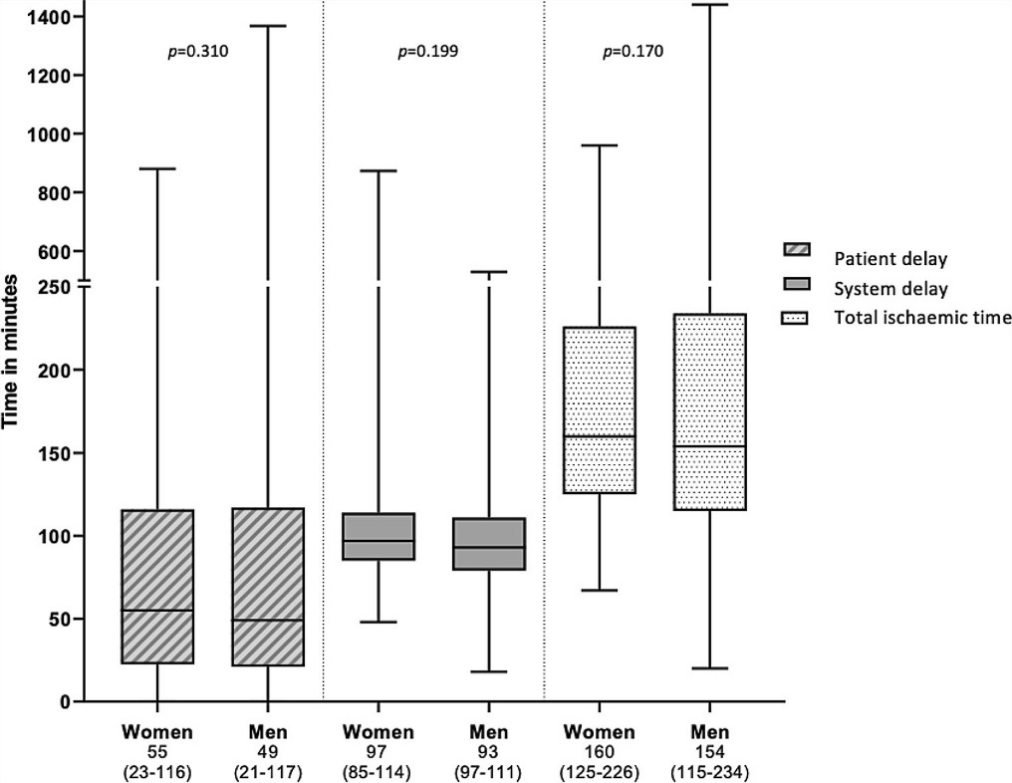
Median time intervals with interquartile range in minutes

**Fig. 2:**
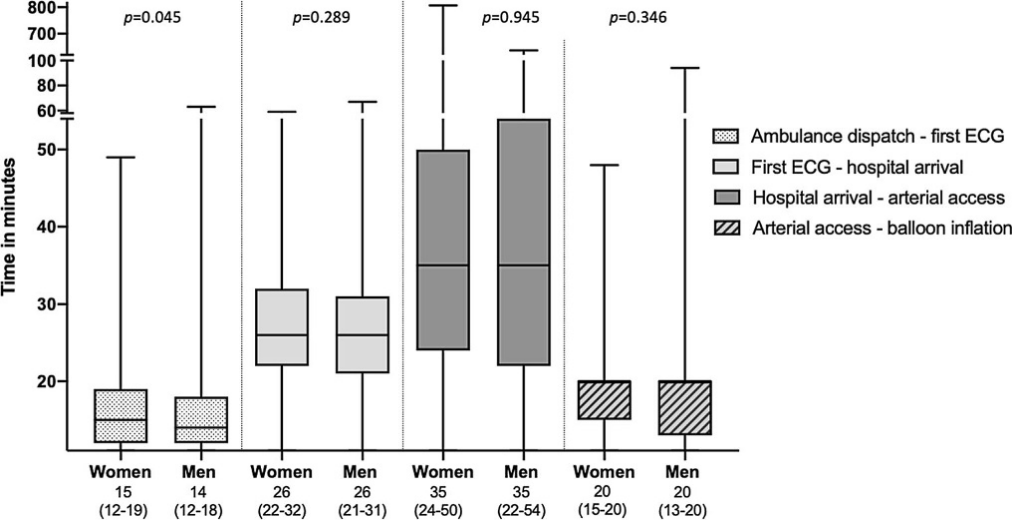
Median time intervals within system delay with interquartile range (*IQR*) in minutes

Tab. 2 and Figs. 1 and 2 present consecutive time intervals from symptom onset until balloon inflation in women and men. No significant differences were found in median patient delay between women and men (55 min vs 49 min, *p* = 0.310). Overall median system delay was not significantly different (97 min in women, 93 min in men, *p* = 0.199). When separating specific time intervals, the time from ambulance dispatch until recording the first ECG was 1 min longer in women (15 min in women vs 14 min in men, *p* = 0.045). Median door-to-arterial access and door-to-balloon times were identical for women and men (35 and 55 min, respectively). Overall median ischaemic time was not statistically significant between sexes (160 min in women, 154 min in men, *p* = 0.170). According to the Dutch ‘*veiligheidsmanagementsysteem*’ (VMS) criteria [14], which excludes the OHCA group, the time from first ECG until arterial access was within 90 min or less in 85% of all patients (87% in women, 85% in men, *p* = 0.416).

**Table 2:** Time intervals from symptom onset to balloon inflation

	Women		Men		Total		*p*-value^b^	*p*-value^c^
	Mean	Median (IQR)	Mean	Median (IQR)	Mean	Median (IQR)		
*Patient delay*								
Symptom onset—ambulance dispatch^a^	107	55 (23–116)	121	49 (21–117)	117	51 (22–117)	0.926	0.310
*System delay*								
Ambulance dispatch—first ECG^a^	17	15 (12–19)	16	14 (12–18)	16	14 (12–18)	0.015	0.045
First electrocardiogram—hospital arrival^a^	27	26 (22–32)	26	26 (21–31)	26	26 (21–31)	0.036	0.289
Hospital arrival—arterial access^a,d^	46	35 (24–50)	43	35 (22–54)	44	35 (23–52)	0.532	0.945
Arterial access—balloon inflation^d^	18	20 (15–20)	18	20 (13–20)	18	20 (14–20)	0.327	0.346
Total system delay^a^	107	97 (85–114)	100	93 (79–111)	102	94 (81–112)	0.168	0.199
*Total ischaemic time*								
Symptom onset—balloon inflation^a^	213	160 (125–226)	218	154 (115–234)	217	157(116–233)	0.571	0.170

*IQR* interquartile range, *ECG* electrocardiogram

^a^Logarithmic transformation was used to calculate the mean

^b^When comparing means by using linear regression

^c^When comparing medians using median test for two independent medians

^d^Hospital arrival—balloon inflation comparable to door-to-balloon time

### Outcome

**Fig. 3:**
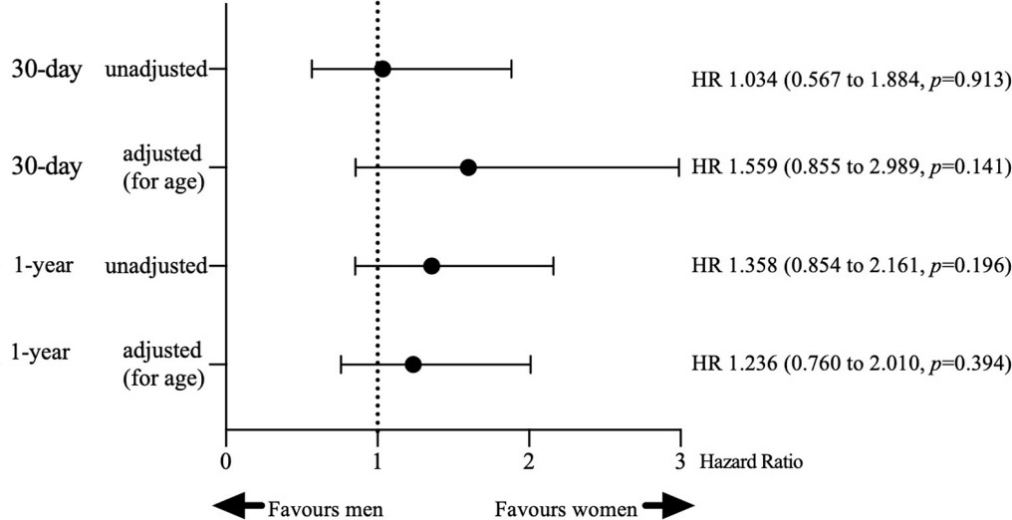
Risk of death (all-cause mortality) after primary percutaneous coronary intervention (*pPCI*)

**Fig. 4:**
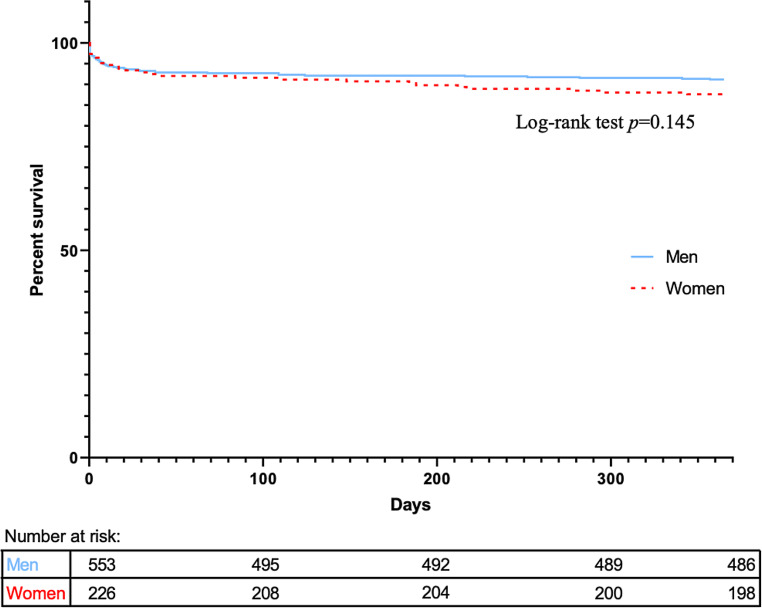
Kaplan Meier curve for 1‑year survival

Thirty-day and 1‑year all-cause mortality hazard ratios are shown in Fig. 3. Thirty-day (both 7%, *p* = 0.913) and 1‑year (12% in women vs 9% in men, *p* = 0.196) mortality rates were not significantly different between women and men. The corresponding Kaplan-Meier survival curve, including numbers at risk and log-rank test, is plotted in Fig. 4. The OHCA group consisted of 84 patients (11%), 19 women and 65 men. Exclusion of the OHCA group did not significantly influence the short- or long-term mortality. Fifteen OHCA patients died within 30 days post-procedure (18%; 3 women, 12 men) and another 3 died within 1 year (4%; 2 women, 1 man).

## Discussion

In this quality registry study, no significant differences were seen when comparing patient delay, system delay and overall ischaemic time between women and men diagnosed with STEMI and receiving pPCI in the Amsterdam region. Regarding 30-day and 1‑year all-cause mortality after pPCI, no significant difference was found between women and men, even after excluding patients with OHCA.

A European multinational registry showed women to be 5% more likely than men (75.9% vs 70.4%) to present to a hospital after more than 120 min from symptom onset [4]. In a French meta-registry, patient delay in women was significantly longer than in men by a mean of 14 min [2]. A Dutch study by Velders et al. [7] showed that women had significantly longer total ischaemic times, with a median difference of 17 min. In our study, we did not find increased patient delay in women. Women and men seem to recognise their symptoms and call for help equally fast. The discrepancy between this current finding and previous research could be explained by the following reasons. Firstly, campaigns focusing on the variety of symptoms suggestive of myocardial ischaemia performed by the Dutch Heart Foundation, such as the yearly Dress Red Day campaigns on 29 September, are considered effective in increasing awareness concerning the variety in presentation between women and men. Next, the positive influence of social and cultural equality regarding the position of women in large cities like Amsterdam could result in women being less disadvantaged. Lastly, general practitioners do not interfere in the STEMI care pathway, leading to less sex-specific bias and possibly resulting in similar delays between women and men. The significant finding showing median time from ambulance dispatch until recording the first ECG to be 1 min longer for women is most likely explained by chance in combination with the relatively small group of women included in the study.

This study shows that ticagrelor was administered in only 82% of cases. The reasons for not administering ticagrelor were often functional, such as presentation with OHCA with inability to take tablets, the unavailability of water or after specific instructions from the cardiologist. It might be of future interest to investigate the effect of administering cangrelor intravenously to this group of patients, as intravenous access is easier in sick patients. Moreover, the use of cangrelor in the CHAMPION PHOENIX trial even showed fewer peri-procedural complications when compared to an oral P2Y_12_ inhibitor [15]. The timing of the procedure (morning/evening/night) did not differ between women and men, which is in line with the results of previous research [16]. Recently, Cencko et al. [4] performed a prospective multinational registry study in 10,443 STEMI patients (3112 women) and showed women were disadvantaged regarding dual antiplatelet and anticoagulant medication administration within 24 h after admission [4]. This finding is in contrast to that of our study, since we found comparable pre-procedural medical treatment in women and men in the Amsterdam region. It could be hypothesised that other European countries might have more difficulty in identifying women with STEMI and treating them accordingly, as they usually present with more diverse symptoms compared to men according to a Swedish multicentre survey study [17].

The all-cause short-and long-term hazard ratio was higher amongst women, yet did not reach statistical significance, which is in contrast to the findings of previous research. In the literature, women present with more explicit cardiovascular risk profiles compared to men [3, 18, 20], which likely explains their elevated early [2, 3, 5, 6, 19, 21] and 1‑year [6, 22] mortality rates following pPCI. Concerning risk factors, the current study reports only on differences in the prevalence of hypertension and smoking status. This variation in risk profiles might explain why women do not show significantly higher death rates than men. Moreover, total ischaemic time did not significantly differ between the two sexes in the current study, whereas contrasting ischaemic times have been reported previously. De Luca et al. [23] showed that every 30 extra minutes of ischaemic time will increase 1‑year mortality by 7.5%. Therefore, mortality rates could have been influenced by increased ischaemic times. However, the most important possible explanation remains the relatively small sample size, which might have resulted in the inability to reach statistical significance.

Countries like France, Sweden and the UK have comparable logistics. Results show that the median total ischaemic time (symptom-to-balloon time) for pPCI for men and women combined is shortest in the UK (230 min) [6], followed by the Netherlands (314 min), Sweden (345 min) [6] and France (385 min) [2]. However, this comparison includes patient delay, which is subjective and ideally would have covered the time span from ECG diagnosis until arterial access/balloon inflation. Also, it must be pointed out that this comparison is only to provide some insight into international acute care and that a direct comparison of results is hard due to heterogeneity of the studies. The Dutch national safety program, the VMS [14], has set a goal regarding STEMI treatment in the Netherlands, stating that arterial access should be achieved within 90 min after STEMI diagnosis in 90% of all patients. According to our current study this goal is achieved in 85% of all patients (with exclusion of the OHCA group), meaning that STEMI management in the Amsterdam region is performing almost to the pre-set goal. The situation in France is different; according to a recent publication on the FAST-MI trial, pPCI was performed within 120 min in only 54% of patients presenting with STEMI [24]. In 28% of the total study population pPCI was performed after 120 min or more, and according to the ESC guidelines a pharmaco-invasive strategy would have been the preferred form of treatment. Patients who present with an OHCA after suffering STEMI have often been excluded from other studies [1, 2, 17] or only included after re-establishment of circulation [7]. However, in this study the OHCA group was not excluded to achieve the main goal, namely comparing STEMI management between women and men. Inclusion of the OHCA group showed 70% of the people are treated within 90 min after diagnosis, an increase of 6% compared to previous data [25]. The use of all-comers data in this study is a realistic reflection of the population within the current situation.

Strengths of this study are the gathering of consecutive patients presenting with STEMI in three pPCI centres in Amsterdam and the surrounding region, performed in a well-coordinated ambulance to pPCI collaboration during 2 years. The main limitation of the present study is the small population compared with the number of patients included in registries such as the SWEDEHEART [3], French [2], European multinational registry [4] or combined registry from the UK and Sweden [6]. It could be hypothesised that the relatively small sample size of the current study influenced the ability to reach statistical significance due to lack of power. Therefore, national registries are important to investigate differences between women and men and to improve treatment strategies and outcome. Due to the study design and including only patients receiving PCI within 24 h, it is very likely that selection bias might have occurred. Due to the lack of data on the cause of death, all-cause mortality was reported instead of the preferable specific cardiovascular mortality.

## Conclusion

Analysing women and men in Amsterdam and the surrounding region receiving pPCI after presenting with STEMI resulted in no differences when comparing patient delay, system delay, overall ischaemic time, medical treatment, 30-day and 1‑year mortality. STEMI management in Amsterdam and the surrounding region performed according to pre-set VMS goals in most patients. The use of the Netherlands Heart Registration to combine data across the country will increase the validity of statements addressing STEMI management in the Netherlands following pPCI. This will even provide insight into outcomes and possible complications and is needed to increase the number of women included, which remains underreported when compared to men.

## Appendix

### Definitions

Hypertension: A systolic blood pressure >140 mm Hg or previous pharmacological treatment
Diabetes mellitus: Both insulin- and non-insulin-dependent diabetes mellitus with a fasting blood glucose >7.0 mmol/l, non-fasting glucose >11.1 mmol/l or previous pharmacological treatment
Hypercholesterolaemia: A total cholesterol of ≥6.5 mmol/l and/or use of lipid-lowering pharmacological treatment
Positive family history: A first-degree family member with occurrence of a cardiovascular event before the age of 60 years
Out-of-hospital cardiac arrest: A loss of effective cardiac activity with absence of circulatory signs needing direct emergency treatment
Severe bleeding: Patients admitted to the hospital due to excessive blood loss and/or receiving one or more units of packed cells
Repeat PCI: Patients receiving PCI in the period after the pPCI
Cerebrovascular event: Both haemorrhagic or an ischaemic cerebrovascular event diagnosed by a doctor
